# Anti‐inflammatory and immune‐mediated therapy for a case of febrile infection‐related epilepsy syndrome with rapid recurrence

**DOI:** 10.1002/ccr3.5952

**Published:** 2022-06-07

**Authors:** Tomonori Kurimoto, Tsuyoshi Matsuoka, Yuki Ami, Koji Kanno, Takashi Fujii, Naoki Fujiwara, Takashi Matsuoka

**Affiliations:** ^1^ Pediatrics Department Okinawa Prefectural Nanbu Medical Center and Children's Medical Center Shimajiri‐gun Okinawa Japan

**Keywords:** FIRES, granzymes B, high‐dose PHB, IL‐6, immune therapy, methylprednisolone, PDCD1, tacrolimus, tocilizumab

## Abstract

Febrile infection‐related epilepsy syndrome (FIRES) is a disease of unknown etiology, characterized by refractory frequent focal seizures, which require prolonged intensive care. We successfully treated a boy with FIRES with anti‐inflammatory and immunosuppressive therapy. This case suggests that an autoimmune mechanism may play a role in the development of FIRES.

## Abstract 2

Immune suppression was effective in a patient with short‐term relapse, suggesting the probability of an immune‐mediated mechanism for Febrile infection‐related epilepsy syndrome.

## 1 INTRODUCTION

Febrile infection‐related epilepsy syndrome (FIRES) is diagnosed by clinical symptoms in patients with new‐onset refractory status epilepticus without a history of active epilepsy or other associated neurological disorders and without definite acute or active structural, toxic, or metabolic causes. Fever begins more than 24 h before the onset of refractory status epilepticus, with or without fever at the onset of status epilepticus.^1^ Ideal treatment for this condition has not yet been determined. We report a case in which tacrolimus was administered after methylprednisolone pulse therapy without exacerbating inflammation, resulting in suppression of seizure.

## 2 CASE REPORT

A previously healthy 13‐year‐old boy had a 3‐day history of fever, deviated gaze to the right, and repeated episodes of generalized tonic–clonic seizures lasting 1–5 min, every 5–10 min. The patient had a medical history of autism spectrum disorder, mild intellectual disability (IQ 63), and obsessive–compulsive disorder. Antiepileptic‐drug (AED) treatment (MDZ and PHB) was initiated, but the seizures increased in frequency; therefore, the patient underwent hypothermia treatment and received thiamylal sodium. Computed tomography scan, urine organic acid analysis, blood microbiological tests, metabolic and cerebrospinal fluid (CSF) analyses, and oligoclonal bands were normal. From the above, the patient was diagnosed as FIRES.^1^

Electroencephalography (EEG) showed an unstructured background tracing with the slow activity of great amplitude and diffuse expression. It registered an autonomic focal seizure with right EEG changes and subsequent generalization. Cranial magnetic resonance imaging (MRI) revealed no abnormalities.

The administration of intravenous immunoglobulin (1 g/kg/day for 2 days), mPSL (methylprednisolone pulse therapy: 1000 mg/day for 3 days), and KD (a high‐fat, low‐protein, low‐carbohydrate diet) did not abate the seizures.

The patient underwent treatment for hypothermia and received thiamylal sodium (4 mg/kg/h), lidocaine (2.5 mg/kg/h), and high‐dose PHB (20–25 mg/kg/day for 14 days), according to a previously published protocol.^2^ Multiple AEDs (valproate sodium, levetiracetam, topiramate, phenytoin) in different combinations were administered under respiratory management. Barbiturates, including phenobarbital, have severe side effects^3^, ^4^; they caused the patient’s serum alanine aminotransferase levels to rise to 1839 U/L. High‐dose PHB was discontinued.

On day 36, after admission, fluid‐attenuated inversion recovery images revealed new hyperintense areas in the hippocampus, amygdala, and claustrum. CSF and blood cytokine levels were elevated, suggesting that a cytokine storm may be involved in the pathogenesis of FIRES. Based on a previous study,^5^ we administered tocilizumab (8 mg/kg/day) two times. The seizures were temporarily suppressed; however, 2 weeks later, the seizures recurred. Therefore, tocilizumab was readministered. The frequency of epileptic seizures increased. On admission, antibodies to N‐methyl‐d‐aspartate type glutamate receptor were found to be high, and intrathecal dexamethasone was administered in accordance with the treatment protocol for FIRES.^6^ IL‐6 in the serum, neopterin and ferritin in the CSF, and β2‐microglobulin/Cre in the urine all showed a decreasing trend.

The seizures worsened again on day 89, and an MRI showed features of edema from the left frontal cortex to the subcortex along with bilateral hippocampal atrophy. Thiamylal administration was resumed.

**FIGURE 1:**
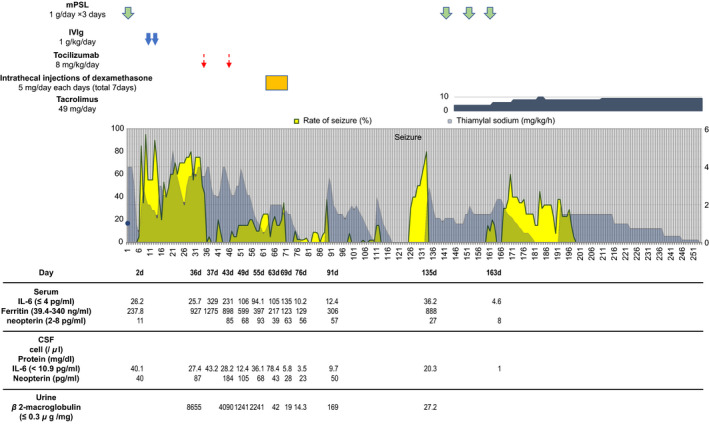
This chart shows the relationship between the frequency of seizures and the dose of thiamylal sodium and other treatments. In addition, changes in markers of blood, cerebrospinal fluid, and urine inflammation during hospitalization have been shown over time

On day 133, MRI showed features of widespread edema extending from the left frontal cortex to the subcortical areas and at the base of the right frontal lobe. The high level of granzyme B (20.9 pg/ml) in the CSF and single‐nucleotide polymorphism of the immunomodulatory gene and programmed cell death protein 1 (PDCD1) were confirmed in the samples collected at admission. Therefore, mPSL pulse therapy (1000 mg/day for 3 days) and tacrolimus (initial dose of 0.1 mg/kg/day) were administered, as per a previously published study.^7^ The seizures were then suppressed, and the patient stabilized (Figure 1).

## 3 DISCUSSION

The pathophysiology of FIRES is poorly understood; however, it may have a paraneoplastic autoimmune origin and may reflect neuroinflammation.^8^ Only a few studies have linked cytokine levels with treatment over time.^9^, ^10^ Few proinflammatory cytokines have been shown to be increased in serum after seizures: however, data on CSF cytokine levels in relation to seizures are not well understood.

IL‐6 induces the differentiation of B cells into antibody‐producing cells and induces the differentiation of naive CD4 T cells into IL‐17‐producing T helper cells (Th 17). The latter induce autoimmune tissue damage and act on CD8 + T cells to induce cytotoxic T cells. Additionally, IL‐6 is produced by monocytes and macrophages with toll‐like receptors.^11^ IL‐6 is commonly found in small amounts in the central nervous system (CNS); however, stimulation of astrocytes and microglia increases the production of IL‐6 in the CNS. Furthermore, increased levels of other cytokines, such as TNF‐α, IL‐Iβ, IFN‐γ, and IL‐17, upregulate IL‐6. Upregulation of IL‐6 reduces hippocampal neurogenesis, increases gliosis, and creates conditions that contribute to epileptogenesis.^12^

TNF‐alpha, IL‐6, and IL‐8 increase the expression of adhesion molecules and activate inflammatory cascades, resulting in neurotoxicity, neuronal excitability, blood–brain barrier (BBB) disruption, and hippocampal damage.^9^

Tocilizumab is a humanized monoclonal antibody against IL‐6 receptors that suppresses the immune effects of IL‐6. Tocilizumab cannot cross the BBB under normal conditions; however, in the presence of BBB dysfunction, it is postulated that it can cross the BBB.^12^ Elevated cytokine levels under prolonged seizure conditions may promote BBB disruption and increase tocilizumab permeability. These results indicate that the increase in IL‐6 levels in the CSF on day 37 may have been due to the effects of tocilizumab on IL‐6R, which decreased the binding of IL‐6 to IL‐6Rs. Moreover, a decrease in IL‐6 levels was achieved by administering tocilizumab and intrathecal dexamethasone.

Tocilizumab therapy and intrathecal dexamethasone were effective, but seizures had relapsed. These were accompanied by the cytokine rise, and the abnormality of the immunomodulating gene was concerned in the prolongation of the inflammatory reaction, as it is proven from the single‐nucleotide polymorphism of the immunomodulatory gene and PDCD1.

The patient was treated with mPSL pulse therapy and continued tacrolimus, and the course was completed without exacerbation of inflammation. The case was caused by autoimmune encephalitis in FIRES. Therefore, it seemed to be necessary to continue the anti‐immune agent use. It was reported that most patients with FIRES gradually recover within 2 months after the onset of their first seizure episode.^13^ This report represents a rare case of rapid recurrence with a progressive course.

Samples collected at the onset of seizures showed elevated granzyme B levels (≥20.9 pg/ml). Moreover, the PDCD1 polymorphism and the efficacy of tacrolimus—an immunosuppressive agent—indicate that an autoimmune mechanism may have contributed to the development of FIRES.^14^ Thus, the pathogenesis of FIRES may involve immune‐mediated encephalitis via the activation of microglia, T cells, and autoantibodies. Future studies need to examine the molecular pathogenesis and clinical course of FIRES to clarify the disease‐state mechanism and develop effective treatment strategies.

## 4 AUTHOR CONTRIBUTIONS

YA, KK, TF, NF, and TaM conceptualized this study. TK conceptualized and designed the study, and wrote and edited the manuscript. TsM supervised the study. All authors approved the final manuscript as submitted and agreed to be accountable for all aspects of the work.

## 6 CONFLICT OF INTEREST

The authors have no conflicts of interest to declare.

7

## 8 ETHICAL APPROVAL

Parents or guardians provided informed consent. Additionally, this case study was conducted in accordance with the provisions of the Declaration of Helsinki, as revised in Tokyo in 2004, and was approved by the Ethics Committee of Okinawa Prefectural Nanbu Medical Center and Children's Medical Center.

## 9 CONSENT

Written informed consent was obtained from the patient to publish this report in accordance with the journal's patient consent policy.

## Data Availability

Data ownership to support the results reported in the manuscript is owned by the institution and controlled by the research representative.
